# Cardiopatia Congênita no Adulto. Modelo para o Seguimento Clínico

**DOI:** 10.36660/abc.20250173

**Published:** 2025-11-13

**Authors:** Fernando Amaral, Geraldo Luiz Figueiredo, Rafael B. Pavão, Paulo Henrique Manso, Luis Gustavo Gali, Danilo Tadao Wada, André Schmidt

**Affiliations:** 1 USP Hospital das Clínicas da Faculdade de Medicina de Ribeirão Preto Ribeirão Preto SP Brasil Unidade de Cardiopatia Congênita no Adulto, Hospital das Clínicas da Faculdade de Medicina de Ribeirão Preto, USP, Ribeirão Preto, SP – Brasil

**Keywords:** Cardiopatias Congênitas, Assistência Ambulatorial, Adulto

Após uma experiência inicial relatada neste Jornal em 2010^1^ e uma análise mais detalhada publicada em 2020,^2^ agora há mais de 1.600 pacientes registrados em nossa clínica ambulatorial. Essa experiência adquirida ao longo de dezoito anos nos permitiu enfrentar diversas situações relacionadas à dinâmica da unidade e aos problemas individuais dos pacientes, as quais acreditamos serem úteis tanto para os responsáveis pelas novas unidades emergentes quanto para as já estabelecidas. Pacientes com cardiopatia congênita em adultos (CCA) apresentam uma prevalência de 4 a 6/1.000 indivíduos,^3,4^ e a maioria deles apresenta o perfil peculiar de uma doença de longa duração, frequentemente iniciada ao nascimento e frequentemente submetida a intervenção cardiovascular. O Dr. Joseph Perloff, em um artigo marcante de 1973,^5^ enfatizou que pacientes com CCA jamais seriam curados e que sequelas e resíduos após tratamento invasivo deveriam ser esperados na maioria deles. De fato, ao longo dos anos subsequentes, estudos demonstraram que a incidência de lesões residuais é quase uniforme, justificando a vigilância de rotina, exceto para uma minoria de indivíduos com lesões muito simples.^6^ Como a alta é rara, se espera que o número de pacientes aumente progressivamente, o que pode interferir no cuidado adequado. Quatro tópicos parecem ser cruciais para nós em relação ao seguimento e são brevemente relembrados a seguir.

## Onde esses pacientes devem ser acompanhados?

**Figura 1:**
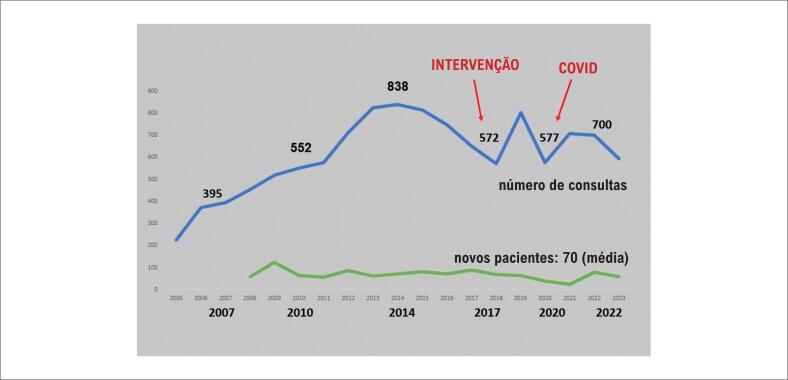
Número de consultas e novos casos durante 18 anos na clínica de CCA do Hospital das Clínicas de Ribeirão Preto. INTERVENÇÃO: tentativa de reduzir o número de consultas de rotina seguindo decisão do grupo e sugestões de diretrizes; COVID: redução significativa no número de consultas durante a pandemia.

Um artigo notável publicado em 2010 mostrou que mais de 90% dos pacientes europeus com CCA não eram acompanhados em uma unidade especializada.^7^ Outros relatórios também divulgaram esse tipo de informação e contribuíram para o necessário maior interesse no manejo desses pacientes, embora muito ainda precise ser feito. Idealmente, todos eles deveriam ser atendidos em uma clínica para adultos de uma unidade terciária multidisciplinar, onde recursos diagnósticos completos, não invasivos e invasivos, estejam disponíveis e a intervenção e cirurgia percutânea sejam realizadas por profissionais com experiência em cardiopatia congênita, os chamados centros de nível I.^8^ Cuidados adequados devem estar disponíveis para tratar gravidez de alto risco, hipertensão pulmonar, insuficiência cardíaca refratária, reabilitação, bem como questões genéticas, cuidados paliativos e psicológicas. Como se espera que a carga de trabalho aumente progressivamente^9^ (Figura 1), se recomenda que pacientes com cardiopatia congênita de complexidade leve sejam acompanhados em unidades secundárias anexas ao centro terciário, permitindo que mais tempo seja dedicado aos casos complexos.^8^ A excelência desse modelo de seguimento, no entanto, nem sempre se verifica. Em muitas regiões de vários países, a probabilidade de uma unidade especializada disponível é pequena, e a assistência geralmente é fornecida em clínicas cardiológicas gerais para adultos.

As clínicas de extensão, onde um cardiologista especializado em CCA visita periodicamente uma unidade secundária, podem reduzir a carga de trabalho dos centros terciários.^10^ Eletrocardiogramas e ecocardiogramas geralmente podem ser obtidos localmente, e investigações adicionais, bem como tratamento invasivo, podem ser realizados no centro terciário. Embora esse padrão de assistência pareça ser uma boa opção, faltam estudos relacionados.

A teleconsulta tem sido utilizada em muitos centros. Embora a interação presencial entre paciente e médico seja uma característica bem reconhecida das boas práticas médicas, a tecnologia pode, pelo menos em parte, superar a ausência desse princípio médico básico, particularmente em indivíduos assintomáticos. Até onde sabemos, protocolos específicos para esse método de consulta não foram desenvolvidos, e as opiniões relatadas se baseiam na experiência e no bom senso do médico.

## Quem deve atender esses pacientes?

O cardiologista ou pediatra responsável pela clínica precisa de profundo conhecimento em cardiopatia congênita. O número de médicos responsáveis pela CCA deve estar relacionado às atividades da unidade e à carga de trabalho de rotina, mas um especialista certificado deve estar sempre disponível.^8^ Algumas unidades em alguns países começaram a oferecer treinamento específico para CCCA^11^ na esperança de preparar o jovem cardiologista ou pediatra.

## Com que frequência esses pacientes devem comparecer à clínica?

Não existe uma regra única. Pacientes com a mesma complexidade de doença podem apresentar quadros clínicos distintos. As diretrizes da CCA publicadas recentemente fornecem recomendações que levam em consideração a complexidade da cardiopatia congênita (I, II, III), que se baseiam nas características anatômicas e, recentemente introduzidas, no estágio fisiológico do paciente (A, B, C, D).^8^ Essa classificação anatômica/fisiológica (AP) permite que pacientes em bom estado clínico sejam acompanhados em intervalos maiores, enquanto aqueles mais gravemente acometidos sejam observados mais de perto em um ambiente menos sobrecarregado. Embora essas medidas possam ser geralmente tomadas e aceitas, se deve enfatizar que a decisão final sobre o intervalo de consulta deve levar em consideração o paciente individual.

**Figura 2:**
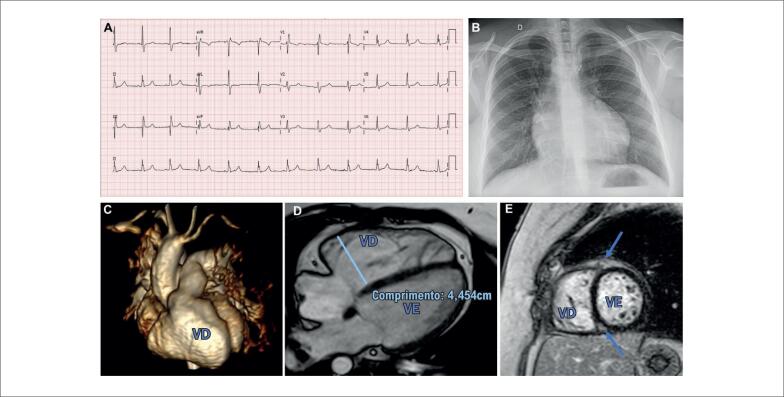
Tetralogia de Fallot. Bom desfecho: mulher, 29 anos, anastomose de Blalock modificada em 4 meses, correção total por atriotomia em 2 anos, assintomática, bloqueio de ramo direito incompleto (BRD) (QRS 110 mseg) e fragmentação ausente no ECG (A), índice cardiotorácico (ICT) 0,58 na radiografia de tórax (B); ressonância magnética cardíaca (RMC): fração de regurgitação pulmonar (FRP) 24%, volumes diastólico final (VDFVD) e sistólico final (VSFVD) indexados do ventrículo direito de 83 e 34 ml, respectivamente, fração de ejeção (FE) ventricular direita e esquerda de 58% e 64%, respectivamente, realce tardio (RTG) (C-E) septal discreto (setas) com gadolínio. Estadiamento AP classificado como IIB. Recomendações de seguimento: consulta/ECG anualmente, HOLTER conforme necessário, RMC/TOMO 2-3 anos.

**Figura 3:**
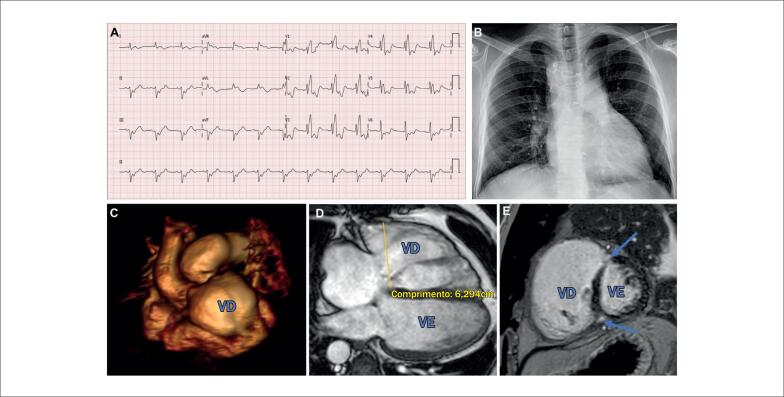
Tetralogia de Fallot. Desfecho comprometido: Homem de 47 anos, correção por ventriculotomia aos 8 anos, substituição da valva pulmonar biológica aos 25 anos, assintomático, BRD (QRS 180 mseg) com fragmentação grave no ECG (A), ICT 0,55 na radiografia de tórax (B), VO_2_ de pico 85% previsto no teste de exercício cardiopulmonar; RMC: VDFVD e VSFVD 183 e 128 ml, respectivamente, FRP 21%, FE ventricular direita e esquerda 30% e 59%, respectivamente, RTG anterosseptal (setas) com discinesia (C-E). Estadiamento AP classificado como IIC. Recomendações de seguimento: consulta de 6 a 12 meses, ECG-ECO anualmente, HOLTER/RMC/TOMO de 1 a 2 anos.

**Figura 4:**
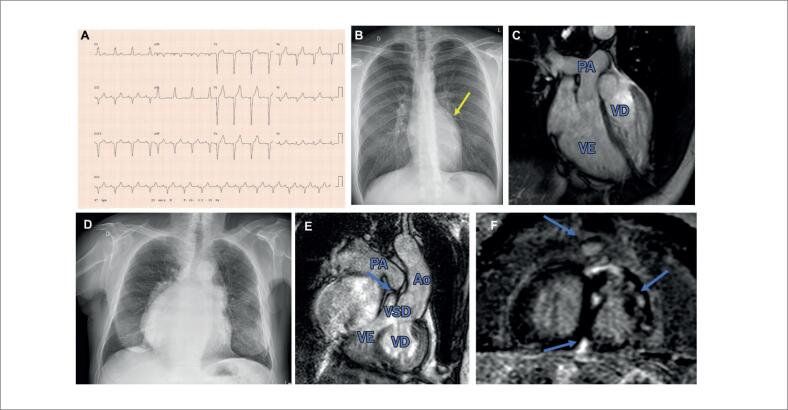
Transposição congênita corrigida das grandes artérias (não operada). **Bom desfecho:** Homem, 31 anos, assintomático, onda q presente em V1 e ausente em V6 (A), coração de tamanho normal mais abaulamento do lado esquerdo (seta) na radiografia de tórax (B); RMC: discordância atrioventricular (AV) e ventrículo-arterial (VA), regurgitação tricúspide (RT) leve/moderada, fração de ejeção ventricular direita e esquerda de 42% e 69%, respectivamente (C). Estágio AP classificado como IIIB. Recomendações de seguimento: consulta, ECG/ECO anualmente, HOLTER: 1-5 anos, RMC/TOMO: 3-5 anos. **Desfecho comprometido:** Homem, 71 anos, recusou cirurgia, classe funcional III em furosemida/espironolactona, dextrocardia/CTI 0,73 na radiografia de tórax (D), ECG/HOLTER fibrilação atrial; RMC: discordância AV/VA, comunicação interventricular perimembranosa de 20 mm, estenose pulmonar grave (seta em E) e regurgitação mitral, FE direita e esquerda de 31% e 39%, respectivamente, realce tardio com gadolínio (setas em F) (E/F). Estadiamento AP classificado como IIID. Recomendações de seguimento: consulta a cada 3-6 meses, ECG/ECO/HOLTER anualmente, RMC/TOMO a cada 1-2 anos.

As figuras mostram exemplos de diferentes políticas em pacientes com as mesmas características anatômicas de complexidade moderada e grave. Como pode ser observado, eles apresentam diferentes estágios fisiológicos relacionados às lesões associadas, tratadas ou não, levando a diferentes recomendações de seguimento (Figuras 2-4).

### Busca ativa

A perda de seguimento é uma situação preocupante na prática médica, ocorrendo também em pacientes com CCA. Sua definição (também relacionada à classificação AP), prevalência, motivos, estratégia de busca e possíveis benefícios foram amplamente discutidos.^12^ Aparentemente, contrariando o necessário processo de alta ambulatorial discutido acima, essa política é recomendada e, em nossa opinião, deve ser considerada uma obrigação do médico responsável.

## Considerações finais

As informações apresentadas acima visam chamar a atenção para alguns aspectos do seguimento ambulatorial da CCA, com foco especial na classificação AP e seu potencial impacto na melhoria do atendimento. Essa prática pode evitar visitas hospitalares desnecessárias para pacientes com bom prognóstico e proporcionar melhor atendimento aos mais acometidos. O número de publicações sobre CCA está aumentando. No entanto, artigos específicos relacionados ao manejo ambulatorial adequado são necessários. As unidades emergentes podem se beneficiar da experiência de unidades mais antigas e oferecer melhor atendimento aos pacientes.

Disponibilidade de Dados

Os conteúdos subjacentes ao texto da pesquisa estão contidos no manuscrito.
