# Supplementary material for: Cardiopatia Congênita no Adulto. Modelo para o Seguimento Clínico
Source: Arq Bras Cardiol. 2025 Nov 13;122(9):e20250173. [Article in Portuguese] doi: 10.36660/abc.20250173 (PMC12671678; doi:10.36660/abc.20250173)
Supplement: Translation

# Translation: Adult Congenital Heart Disease. Follow-Up Pattern of Care

**DOI:** 10.36660/abc.20250173i

**Authors:** Fernando Amaral, Geraldo Luiz Figueiredo, Rafael B. Pavão, Paulo Henrique Manso, Luis Gustavo Gali, Danilo Tadao Wada, André Schmidt

**Affiliations:** 1 Ribeirão Preto SP Brazil Unidade de Cardiopatia Congênita no Adulto, Hospital das Clínicas da Faculdade de Medicina de Ribeirão Preto, USP, Ribeirão Preto, SP – Brazil

**Keywords:** Congenital Heart Defects, Assistência Ambulatorial, Adult

After an early experience reported in this Journal in 2010^1^ and a more detailed analysis published in 2020,^2^ there are now over 1600 patients registered in our outpatient clinic. This acquired eighteen years’ experience allowed us to face several situations related to the unit dynamics and to individual patient problems, which we believe might be useful for those particularly in charge of the new emerging unities and also for those already established. Adult congenital heart disease (ACHD) patients have a prevalence of 4 to 6/1.000 individuals^3,4^ and most of them have the peculiar profile of a long-standing disease frequently started at birth and often submitted to a cardiovascular intervention. Dr Joseph Perloff, in a landmark article in 1973,^5^ emphasized that ACHD patients would never be cured and sequelae and residua after invasive treatment should be expected in most of them. In fact, over the subsequent years, studies have shown that the incidence of residual lesions is almost uniform, justifying routine surveillance, except for a minority of individuals with very simple lesions.^6^ As discharge is rare, the number of patients is expected to increase progressively, which might interfere with proper care. Four topics seem to be crucial for us regarding follow-up and are briefly reminded below.

## Where should these patients be followed up?

A remarkable article published in 2010 showed that more than 90% of ACHD European patients were not followed in a specialized unit.^7^ Other reports have also released this sort of information and have contributed to the necessary greater interest in the management of these patients, although much has yet to be done. Ideally, they should all be seen in an adult clinic of a multidisciplinary tertiary unit where full non-invasive and invasive diagnostic resources are available and percutaneous intervention and surgery are provided by practitioners with CHD expertise, the so-called level I centers.^8^ Appropriate care must be available to address high-risk pregnancy, pulmonary hypertension, refractory heart failure, rehabilitation, as well as genetics, palliative care, and psychological issues. As workload is expected to increase progressively^9^ (Figure 1), it has been recommended that patients with CHD of mild complexity be followed in secondary unities attached to the tertiary center, allowing more time to be devoted to the complex cases.^8^ The excellence of this pattern of follow-up, however, is not always the case. In many regions of several countries, the likelihood of a specialized unit available is small, and assistance is usually provided in general adult cardiological clinics.

**Figure 1:** Number of patient visits and new cases during 18 years at the Hospital das Clínicas de Ribeirão Preto ACHD clinic. INTERVENTION: attempt to reduce the number of routine visits following group decision and guidelines suggestions; COVID: significant decrease in consultation numbers during the pandemic.

The outreach clinics, where a specialized ACHD cardiologist periodically visits a secondary unit, may reduce the tertiary centers’ workload.^10^ Electrocardiograms and echocardiograms can usually be locally obtained, and additional investigation, as well as invasive treatment, can be done at the tertiary center. Although this pattern of assistance seems to be a good option, related studies are lacking.

Teleconsultation has been employed in many centers. Albeit face to face patient-doctor interaction is a well-recognized characteristic of good medical practice, technology may at least in part overcome the absence of this basic medical principle, particularly in the asymptomatic individual. As far as we know, specific protocols regarding this consultation method have not been developed, and reported opinions are based on the physician's experience and common sense.

## Who should see these patients?

Deep CHD knowledge is required for either the cardiologist or pediatrician in charge at the clinic. The number of ACHD physicians in charge should be related to the unit activities and routine workload, but a board-certified specialist should always be available.^8^ A few unities in a few countries started to provide ACHD-specific training,^11^ hoping to prepare the young cardiologist or pediatrician.

## How often should these patients attend the clinic?

There is not a single rule. Patients with the same disease complexity may present distinct clinical pictures. The recently published ACHD guidelines provide recommendations taking into consideration the complexity of the CHD (I, II, III), which is based on the anatomical features and, newly introduced, the patient's physiologic stage (A, B, C, D).^8^ This anatomic/physiologic (AP) classification allows patients in good clinical status to be followed at greater intervals, while those more severely afflicted to be seen more closely in a setting not so overloaded. Although these measures can be generally taken and accepted, it should be emphasized that the final decision regarding the consultation interval should take into consideration the individual patient.

The figures show examples of different policies in patients with the same anatomical features of moderate and severe complexity. As can be seen, they have different physiological stages related to treated or not treated associated lesions, leading to different follow-up recommendations (Figures 2-4).

**Figure 2:** Tetralogy of Fallot. *Good outcome*: female, 29-year-old, modified Blalock anastomosis at 4 months, total correction by atriotomy at 2 years, asymptomatic, incomplete right bundle branch block (RBBB) (QRS 110 mseg) and absent fragmentation on the EKG (A), cardiothoracic index (CTI) 0.58 on chest X-ray (B); cardiac magnetic resonance (CMR): pulmonary regurgitant fraction (PRF) 24%, indexed right ventricular end-diastolic (IRVEDV) and end-systolic (IRVESV) volumes 83 and 34 ml respectively, ventricular right and left ejection fraction (EF) 58% and 64% respectively, discrete (arrows) septal late gadolinium enhancement (LGE) (C-E). AP stage rated IIB. Follow-up recommendations: consultation/EKG yearly, HOLTER as needed, CMR/TOMO 2-3 years.

**Figure 3:** Tetralogy of Fallot. *Impaired outcome*: male 47-year-old, correction by ventriculotomy at 8 years, biological pulmonary valve replacement age 25 years, asymptomatic, RBBB (QRS 180 mseg) with severe fragmentation on EKG (A), CTI 0.55 on chest X-ray (B), peak VO2 85% predicted on cardiopulmonary exercise test; CMR: IRVEDV and IRVESV 183 and 128 ml respectively, PRF 21%, ventricular right and left EF 30% and 59% respectively, antero-septal LGE (arrows) with dyskinesia (C-E). AP stage rated IIC. Follow-up recommendations: consultation 6-12 months, EKG-ECHO every year, HOLTER/CMR/TOMO 1-2 years.

**Figure 4:** Congenitally corrected transposition of the great arteries (not operated). ***Good outcome***: male, 31-year-old, asymptomatic, q wave present in V1 and absent in V6 (A), normal size heart plus left sided bulge (arrow) on chest X-ray (B); CMR: atrioventricular (AV) and ventriculoarterial (VA) discordance, mild/moderate tricuspid regurgitation (TR), ventricular right and left ejection fraction 42% and 69% respectively (C). AP stage rated IIIB. Follow-up recommendations: consultation, EKG/ECHO yearly, HOLTER: 1-5 years, CMR/TOMO: 3-5 years. ***Impaired outcome***: male, 71-year-old refused surgery, functional class III on furosemide/spironolactone, dextrocardia/CTI 0.73 on chest X-ray (D), EKG/HOLTER atrial fibrillation; CMR: AV/VA discordance, 20 mm perimembranous ventricular septal defect, severe pulmonary stenosis (arrow in E) and mitral regurgitation, right and left EF 31% and 39% respectively, late gadolinium enhancement (arrows in F) (E/F). AP stage rated IIID. Follow-up recommendations: consultation every 3-6 months, EKG/ECHO/HOLTER yearly, CMR/TOMO 1-2 years.

### Active search

Loss of follow-up is a worrisome situation in medical practice, also occurring in ACHD patients. Its definition (also related to the AP classification), prevalence, reasons, search strategy, and possible benefits have been well discussed.^12^ Apparently, going against the necessary outpatient unloading process discussed above, this policy is recommended and, in our view, should be considered a physician in charge obligation.

## Final considerations

The information presented above was meant to draw attention to some aspects of ACHD outpatient follow-up with a special focus on the AP classification and its potential impact on better care. This practice may avoid unnecessary hospital visits for patients with a good outcome and will provide better care to the more afflicted ones. The number of ACHD publications is increasing. However, specific articles related to adequate outpatient management are needed. The emerging unities may benefit from older units’ experience and provide better patient care.

Data Availability

The underlying content of the research text is contained within the manuscript.
